# “Ready for Landing”—study protocol for the evaluation of a brief sleep hygiene group intervention for youth with psychiatric disorders

**DOI:** 10.3389/fpsyg.2025.1543448

**Published:** 2025-08-14

**Authors:** Paula Theresa Meyer, Hannah Brauer, Clara Marie Schreiber, Pia Muriel Heinze, Julia Witte, Christoph Berger, Manuel Munz, Alexander Dück, Olaf Reis, Michael Kölch, Alexander Prehn-Kristensen

**Affiliations:** ^1^Institute of Child and Adolescent Psychiatry, Center for Integrative Psychiatry, School of Medicine, Christian-Albrecht University Kiel, Kiel, Germany; ^2^Clinic for Child and Adolescent Psychiatry, Psychotherapy and Psychosomatics, Center for Integrative Psychiatry, School of Medicine, Kiel, Germany; ^3^Department for Child and Adolescent Psychiatry and Neurology, Rostock University Medical Center, Rostock, Germany; ^4^German Center for Child and Adolescent Health (DZKJ), Partner Site Greifswald/Rostock, Site Rostock, Rostock, Germany; ^5^Department of Psychology, Faculty of Human Sciences, MSH Medical School Hamburg - University of Applied Sciences and Medical University, Hamburg, Germany

**Keywords:** child and adolescent psychiatry, sleep hygiene, sleep quality, sleep duration, psychiatric disorders

## Abstract

**Background:**

Many children and adolescents with psychiatric disorders struggle with sleep problems that oftentimes stay unaddressed in therapeutic settings due to limited resources. Still, there is evidence that improving insufficient sleep positively affects mental health recovery. Addressing an adequate sleep hygiene is named to be the first line of treatment when it comes to unspecific sleep problems. The present study aims to evaluate the efficacy of a brief sleep hygiene group intervention for children and adolescents with psychiatric disorders named “Ready for Landing.” The intervention is designed to improve sleep quality and elongate sleep duration by explaining the process of falling asleep using the metaphor of an airplane landing. Elements of psychoeducation and cognitive behavioral therapy for insomnia are combined.

**Method:**

The study plans to investigate the efficacy of the intervention using a waitlist control design. A sample of children and adolescents (aged 10–18 years) that are undergoing treatment for psychiatric disorders in day treatment centers in Kiel and Rostock, Germany, will be included. Sleep quality is assessed using the Pittsburgh Sleep Quality Index. Sleep duration is assessed by using a sleep diary completed by the patients and as well using actigraphy as an objective measure. Analysis of variance will be used to detect treatment effects. Secondary outcomes include variables regarding sleepiness.

**Discussion:**

If “Ready for Landing” proves to be efficacious the intervention has the potential to bridge the gap between frequent sleep problems in youth with mental health problems and limited resources to target those. Serving as an effective and economic tool, it could support mental healthcare professionals. It is a promising intervention to support the positive outcome of mental health treatments by improving sleep in children and adolescents.

**Clinical trial registration:**

https://www.bfarm.de/DE/Das-BfArM/Aufgaben/Deutsches-Register-Klinischer-Studien/_node.html, identifier DRKS00034984.

## 1 Introduction

Sleep disturbances and dissatisfactory sleep are highly prevalent in individuals with mental health problems. For example, in children with autism spectrum disorder, a prevalence for sleep problems ranging from 60 to 86% is reported (Souders et al., 2017). In ADHD, an estimated 25–50% of patients suffer from sleep disturbances (Al Lihabi, 2023) while other studies report prevalence rates up to 70% (Martin et al., 2020; Loram et al., 2023). Regarding mood disorders, Asarnow and Mirchandaney (2021) reported that among adolescents with major depression, the prevalence of sleep disturbances ranges from 33 to 72.7%, depending on age and the specific definition used. For children and adolescents suffering from bipolar disorder, estimates range from 21 to 82%. In children and adolescents with anxiety disorders, parents of 85% of the participants reported sleep disturbances, while 52% of the youth themselves indicated trouble sleeping. Given the variety of disorders with cooccurring sleep difficulties those are mostly considered unspecific, transdiagnostic symptoms and are sometimes regarded as secondary due to their high prevalence (Freeman et al., 2020). This is concerning, as sleep disturbances might be a risk factor for the development (Reynolds and Alfano, 2016) and maintenance (Baglioni et al., 2011; Baglioni et al., 2016; Inada et al., 2021) of psychiatric disorders and detrimental (Inada et al., 2021) to the effect of therapeutic interventions.

The sleep of children and adolescents undergoes significant changes during puberty, which can further increase the risk to develop sleep disorders and may contribute to the onset or worsening of other psychiatric disorders (Tarokh et al., 2014; Reynolds and Alfano, 2016). For children and adolescents who are already affected by mental health conditions, research indicates rising strain from sleep problems in parallel to the severity of those mental health concerns (Boafo et al., 2021). Accordingly, improvement in sleep hygiene can be seen as a first line treatment or prevention to reduce the impact insufficient sleep has on those with mental disorders. It has proven effective in the treatment of various mental health conditions (Freeman et al., 2020; Tarokh et al., 2014; Gupta et al., 2019; van der Heijden et al., 2018; Boafo et al., 2021; Inada et al., 2021; Hertenstein et al., 2022). In adults treatment guidelines recommend sleep hygiene as part of Cognitive Behavioral Therapy for Insomnia (CBT-I) as first line and gold standard treatment (Hertenstein et al., 2022). Sleep hygiene mostly incorporates behavioral modification and lifestyle based interventions that improve both sleep quality and quantity and promote better sleep (Baranwal et al., 2023). The need for accessible and effective interventions targeting sleep and sleep hygiene is further highlighted in a study by Quon et al. (2018) which found that nearly one-third of children and 71% of adolescents visiting a mental health outpatient center had poor sleep quality. The results furthermore indicated, that when focusing on specific domains of insufficient sleep quality, 88% of the children and 97% of the adolescents showed a problem in at least one of the seven domains assessed by the Pittsburgh Sleep Quality Index (PSQI; Buysse et al., 1989). The need for those interventions has led to the development of a variety of interventions, as for example F.E.R.R.E.T (Tan et al., 2012), which targets different domains of behavioral and cognitive modification strategies and is suitable for various patient-and age groups, or TransC-Youth (Harvey et al., 2025), which was evaluated for the use in an individual and group therapy community setting. So far, there is a high variety of interventions, which need to be further developed but also critically evaluated with high-quality evaluative studies (McLay et al., 2024; Hall and Nethery, 2019; Chung et al., 2018; Nikles et al., 2020). It hast to be noted that stand-alone sleep hygiene interventions, when not implemented as part of CBT-I, are not the recommended gold-standard treatment for diagnosed insomnia (Deutsche Gesellschaft für Kinder-und Jugendpsychiatrie, Psychosomatik und Psychotherapie e.V. (DGKJP), 2018). However, they are recommended as a first line treatment for children and adolescents with psychiatric disorders that present with comorbid sleep disturbances (van der Heijden et al., 2018; Tarokh et al., 2014). To account for highly prevalent sleep problems and to address the need for easily implementable interventions we developed a group intervention focused on sleep hygiene that includes psychoeducation as well as some few easy-to-learn elements of cognitive behavioral therapy for insomnia. Since most of the participants have not been diagnosed with insomnia, but suffer from sleep problems nevertheless, the intervention addresses their need for improved sleep. The group intervention uses a metaphorical framework, linking the principles of sleep hygiene to pictorial language to help children and adolescents memorize the rules.

In this context, the intervention “Ready for Landing” (RfL) presented in this protocol explains the principles of sleep hygiene by using a metaphor of a landing passenger plane. It uses psychoeducation as well as elements based in CBT-I, such as problem-solving strategies and strategies to overcome barriers in establishing new behaviors (Schlarb, 2015). This aims to convey knowledge while also empowering participants with tools to use it. We intentionally do not incorporate elements such as bedtime restriction. Despite being highly efficacious, these require closer supervision and elongated time for treatment than what is feasible in brief group-therapy sessions or during patient one-on-one therapeutic contacts focusing on primary mental health concerns. We strongly believe the presented combination of psychoeducation with CBT-I strategies therefor offers an accessible, time-efficient way to address common co-occurring sleep problems in mental conditions that led to the admission. Further implemented elements include explanations regarding the functionality of sleep and the development of behavioral intentions and coping mechanisms to overcome potential obstacles when implementing new sleep hygiene routines. Those techniques have been shown to improve sleep quality, sleep hygiene and overall wellbeing, for example, by restructuring or limiting negative thought processes concerning sleep, applying stimulus control or working with sleep related beliefs and the theory of planned behavior (Lin et al., 2018; Meltzer and Mindell, 2014; Chung et al., 2018).

### 1.1 Aim of the study and hypotheses

The present study aims to evaluate the efficacy of a group therapy-based sleep hygiene intervention within day clinic treatment for children and adolescents with psychiatric disorders. We expect to improve sleep quality and elongate sleep duration in children and adolescents with mental health problems by implementing “Ready for Landing” (RfL). On an exploratory level we aim to investigate the intervention’s effect on the sleep onset latency and sleepiness. An additional aim of this study is to investigate the circadian rhythm with regard to its stability and variability, as well as the relationship between subjective (sleep diary and questionnaires) and objective data (actigraphy) on sleep quantity and quality. For this aim an additional study protocol will follow.

## 2 Methods and analysis

### 2.1 Study design

**Figure 1:**
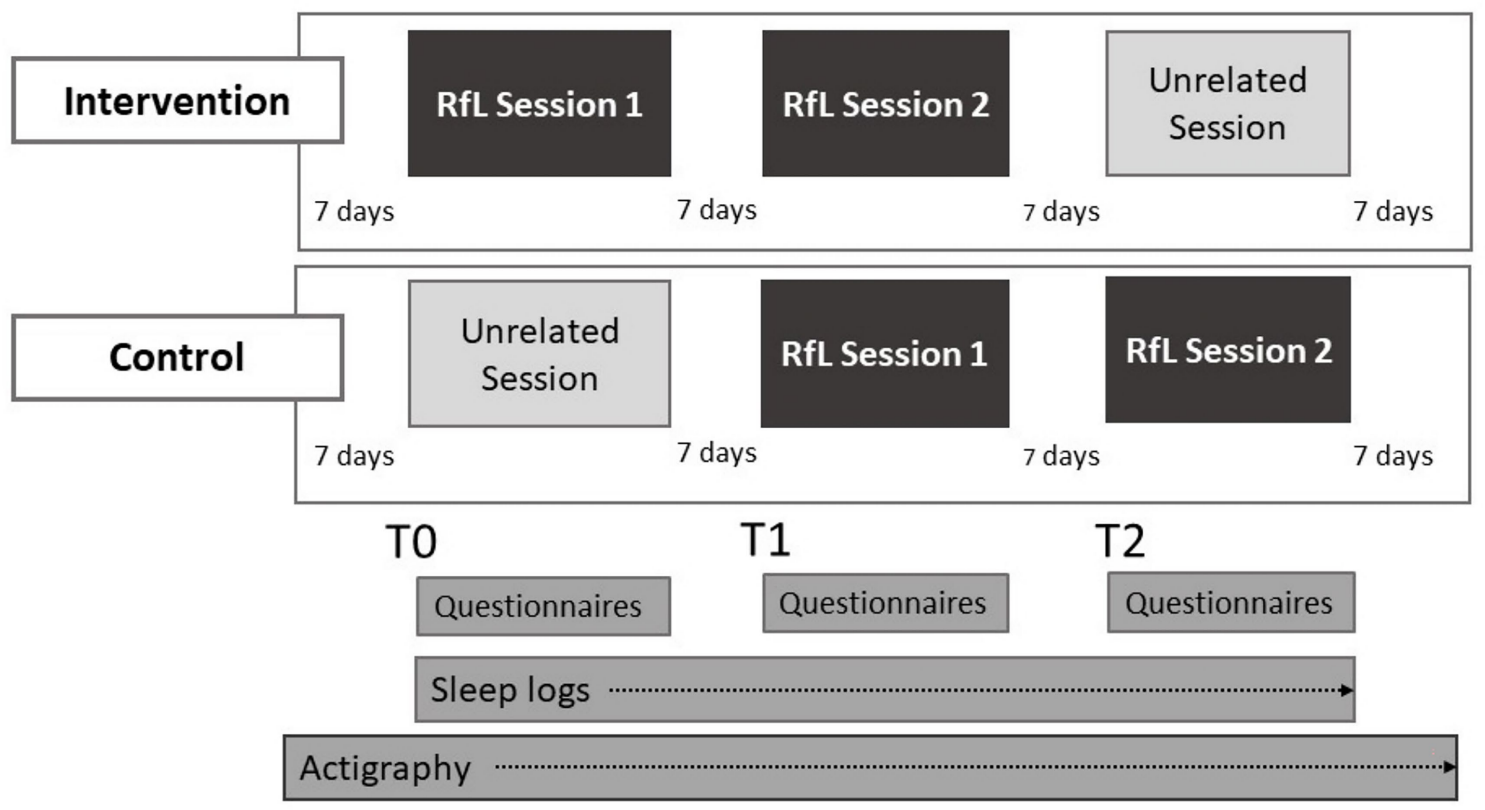
Study design “Ready for Landing” (RfL). Participation consists of 3 sessions in both conditions. The intervention group starts with the two RfL sessions and finishes with intervention-unrelated group therapy while the control group starts with the intervention-unrelated group therapy and then receives the RfL intervention. Intervention and control group are conducted in consecutive, alternating turns separated by an 8–10 weeks pause interval.

The study is conducted using a waitlist-control-design. Each participant attends three sessions in a group therapy setting, with session T0 and T1 forming the primary focus to detect a treatment effect (Figure 1). The third timepoint, T2, serves for further exploratory analysis and to deliver the intervention to the control group as well. Two out of three sessions are the RfL-sessions, while a third one is of an unrelated topic. Participants are quasi-randomized into intervention or control group. Allocation of participants to the group is based on the time they begin treatment in the facility. Whether the participants belong to the control or intervention group is determined by the alternating nature of their occurrence. After an intervention group lasting 28 days an about 8–10 weeks pause interval follows to reach a sufficient amount of new, naive-to-the treatment admissions. After that a control group of 28 days starts, also followed by an 8–10 weeks pause interval afterwards. This process, alternating between intervention and control group with an 8–10 weeks pause interval in between, is continued until the required number of participants has been reached. Participation from start to end lasts 28 days representing 4 weeks around the described timepoints. The measurements collected for this study consist of different self-report questionnaires, sleep logs and actigraphy data. Psychometric properties of the questionnaires used are described below.

#### 2.1.1 “Ready for Landing”—the intervention

“Ready for Landing” (RfL) is conceptualized as a brief, group therapy-based intervention that teaches the principles of sleep hygiene through the metaphor of a passenger plane landing. The intervention is administered by mental health professionals, including psychotherapists, psychologists and social workers. The intervention follows a manual and is therefore standardized in its implementation. All mental health professionals involved receive training on the regimen of the study and the manual of the intervention to ensure quality standards are met.

**Figure 2:**
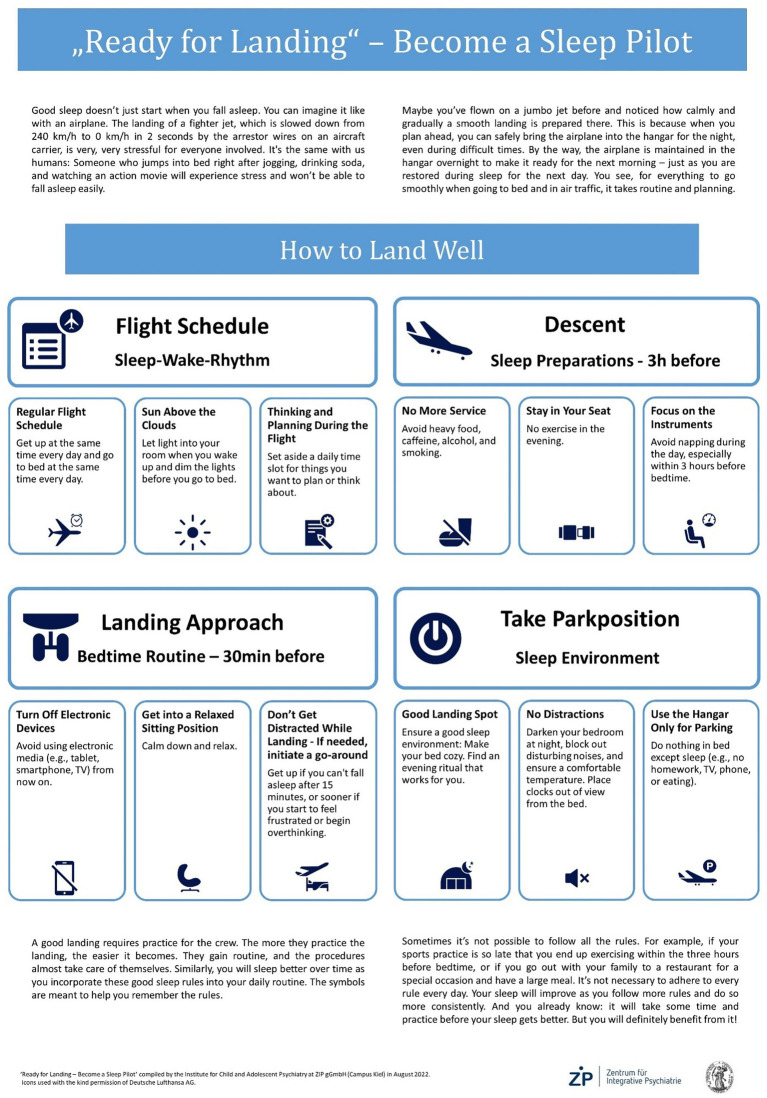
“Ready for Landing”-metaphor. Poster illustrating the airplane metaphor used for the intervention “Ready for Landing”: The poster is hung up between session 1 and 2 and used to explain the sleep hygiene rules during the sessions. The rules are also printed on cards that are used during the intervention.

The intervention consists of two 90-min sessions held 1 week apart from each other. In the intervention group those sessions take place at T0 and T1, the control group undergoes the intervention at T1 and T2. To give patients as much control as possible over their sleep routine, the intervention is implemented in day-clinic treatment settings for children and adolescents in psychiatric care. During the first session, patients learn about the functionality of healthy sleep, reflect on their own current sleep quality, sleep habits, and sleep hygiene and are introduced to the airplane metaphor. The metaphor explains sleep hygiene as a well-structured and segmented process consisting of different phases which enable pilots to land the plane safely, in other words to establish a good routine to go to sleep and is depicted in Figure 2. The landing of a passenger airplane is described to pass through 12 stages which are grouped into four phases: flight schedule, descent, landing approach and park position. In the “flight schedule” phase, consistent times for departure, as well as preparations regarding the takeoff and the flight itself are outlined. During “descent” service of food and beverages is stopped and passengers must remain seated. The patients are further advised not to take naps 3 h before bedtime. According to the common sleep hygiene recommendations it is emphasized not to nap after 3 p.m. The difficulty, however, was that the day clinic treatment was not finished until around three o’clock. In order to address this important rule, therefore the time window had to be adjusted accordingly (no nap 3 h before bedtime). Although the time stated on the poster may seem too late, it was pointed out during training that it was important not to nap too late and certainly not 3 h before bedtime.” During the third phase, the “landing approach,” electronic devices have to be turned off (“flight mode”), passengers are told to sit back and relax and in case of turbulences or obstructions on the runway a loop is flown and landing is restarted shortly after. The fourth phase mainly targets the processes occurring when the plane landed and is pulled into its hangar. It is therefore named “Take park position” and describes the importance of a clean and functional but also welcoming hangar which is used for this purpose only. Each phase corresponds to a specific time window. The first phase mainly describes the day starting with getting up and ending with the time to start preparing for bedtime. The second phase, “descent,” covers 3 h before bedtime while the third phase, “landing approach,” comprises of the last 30 min before the planned bedtime. Phase four, “Take park position,” mainly consist of the night and daytime preparations making sure the sleeping place is ready when it needs to be.

After the introduction patients are told that mastering a smooth landing requires practice and it might also be helpful to practice one step at the time. They are encouraged to reflect their own current ability to “land” using a worksheet asking them about their current sleep hygiene. Afterwards they select one or two sleep hygiene rules at max to practice for the upcoming week. The group is guided to discuss possible obstacles and solutions targeting them to master the selected behavior. The second session consists of a free or cued recall of the 12 sleep hygiene rules depending on the group’s ability to memorize the rules in the beginning of the session. This is followed by a reflection of the patients’ “pilot training” over the last week. Patients are encouraged to continue working on their ability to land smoothly and are reminded that this process might take some time to be mastered. The participants are then awarded their pilot license when being able to correctly answer questions about the newly learned sleep hygiene rules in a quiz. At the end of the session patients can ask further questions about sleep and sleep hygiene and are awarded with a “flight tag” to thank them for their participation and to serve as a reminder for the newly learned principles.

#### 2.1.2 Questionnaires and actigraphy

The study aims to detect improvement in subjective and objective domains of sleep following the intervention. Subjective sleep quality is assessed using PSQI (Buysse et al., 1989). The PSQI consists of 24 items covering seven domains of sleep quality including sleep quality, sleep latency, sleep duration, sleep efficiency, sleep disturbances, use of medication, and daytime sleepiness. The PSQI is a validated and widely used self-report measure for sleep quality showing good reliability, internal consistency, criterion validity and discriminatory power. A global score of five has been deemed sufficient to discriminate between good and bad sleep quality (Buysse et al., 1989). Those findings have been replicated for the use of the PSQI in minors (La Vega et al., 2015; Larche et al., 2021). To assess daytime sleepiness, we use the Epworth Sleepiness Scale for Children and Adolescents (ESS-CHAD, Janssen et al., 2017) which consists of eight items regarding the tendency to fall asleep during different age-adjusted daytime activities such as during lessons in school. The ESS-CHAD has been shown to be a reliable and valid measure to assess daytime sleepiness in minors (Janssen et al., 2017). Both questionnaires were chosen because they are applicable to our wide age range from 10 to 18 years of age likewise and are commonly used in sleep research. This ensures comparability within the group of participants and also allows to compare our results to those of other researchers. PSQI and ESS-CHAD are distributed at the beginning of every session so that the participants answer them before they receive any new elements of the intervention during the sessions. They are instructed to focus on symptoms present in the last week. Between sessions the patients keep a sleep log that monitors aspects such as bedtime, waketime, total sleep duration, duration until they fall asleep and subjective aspects such as perceived rest-and wakefulness each day during the week. Those subjective measures will be complemented by actigraph data to gain insides about possible incremental validity. The comparability of subjective and objective sleep data collected in this study will be subject of another study. Hence, this will be described in an additional study protocol, which aims to gain insight on subjective and objective measurements of sleep in youth with mental health problems.

In this study actigraphy will only be used in terms of assessing treatment effects on sleep duration (Total Sleep Time, TST). Therefor the MotionWatch 8 (Alpha Trace Ltd.) a wrist worn actigraphy watch worn continuously throughout the 28 day will be used. We will use MotionWatch Mode 1 and record 30 s epochs of movement and light information. Further analysis of actigraphy data such as sleep efficiency and the stability of sleep–wake-rhythm and all efforts to compare subjective and objective sleep data will be described in the additional protocol mentioned above.

At the end of the intervention participants provide feedback about perceived usefulness of the metaphor and materials, adherence during the intervention, intention to further adhere to the introduced behavior and further annotations, positive and negative. We further collect sociodemographic variables such as age, gender, living situation and completed and intended education, as well as current psychiatric disorders (ICD-10 diagnoses) and mental health problems. With this we intend to gain insight into the usefulness of the intervention across various psychiatric disorders and their respective symptom severity on an exploratory level.

### 2.2 Study sample and setting

The study is conducted as a multicenter-study in three day clinics for children and adolescent psychiatry in northern Germany. Participating clinics comprise of the day clinic facility of the Clinic for Child and Adolescent Psychiatry, Psychotherapy and Psychosomatics at the Center for Integrative Psychiatry, School of Medicine in Kiel and the two day clinics of the Department for Child and Adolescent Psychiatry and Neurology at the Rostock University Medical Center in Rostock. All patients and parents/custodians receive age-appropriate information about the study procedure and the collection of data. The collection of study data is only possible if written informed consent is granted by both, children and parents/custodians. Depending on the facility, groups consist of different age ranges overall ranging from 10 to 18 years. Age-related differences regarding time needed to fill out questionnaires and worksheets are accounted for by personnel to ensure the programs practicability across all age groups. Groups may vary in size depending current treatment capacity. Inclusion criteria for this study are rather limited: To take part in the study the patients need to be between 10 and 18 years old, being treated in the facility consistently for the whole duration of the study and need to be able to comprehend presented concepts such as time. Further a sufficient understanding of the German language as well as the ability to read and write short phrases is required. Patients that do not consent to their data being processed take part in the intervention nevertheless. They fill out the questionnaires and sleep logs relevant to the intervention, but those will not be collected for analysis and no actigraphy will be recorded.

### 2.3 Eligibility criteria

There is no specific group of patients with specified disorders targeted in this study since we aim to improve sleep problems across different psychiatric disorders. Participants will be included in the study if they are patients of day clinic facilities of clinics of child-and adolescent psychiatry at the age of 10–18 years old, have sufficient German language skills and gave their written informed consent. Patients at the age under 18 years old will be included if both the patient and legal guardians consented. Participants will be excluded from the study if they leave the inpatient facility before the second intervention session.

### 2.4 Sample size calculation

The sample size was computed assuming a repeated measures ANOVA with a within-between interaction using g*Power 3.1 (Faul et al., 2009) with the sleep duration used as primary outcome measure. We assumed a middle effect size (*d* = 0.25), a power of 0.90 and an alpha-error of 5%. This resulted in a minimum sample size of *N* = 46 participants. As we expect a drop-out rate of about 25%, the target enrollment is 60 participants.

### 2.5 Randomization

Intervention and control group run in alternating turns. Patients are assigned to the condition and undergo the study procedure as scheduled for the condition as soon as enough patients were admitted to the day patient department. After one group completes the intervention, a break of 8–10 weeks follows, after which the other group begins (i.e., after an intervention group and the described break a control group follows) Therefor participants are quasi-randomized into their assigned condition. This process is repeated until the estimated sample size is met.

### 2.6 Blinding

Patients and staff are not blind to the treatment condition.

### 2.7 Outcome measures

Primary outcome measures comprise of subjective sleep quality by using the PSQI and sleep duration measured by actigraphy recordings. To assess changes in subjective sleep quality the PSQI global value is computed as proposed by the authors (Buysse et al., 1989). Sleep duration is measured by using sleep diaries completed by the participants and by monitoring TST with actigraphy.

Secondary outcome measures are composed of daytime sleepiness, which is quantified as the global score of the ESS-CHAD (Janssen et al., 2017). Further actigraphy dependent outcome measures are outlined in an additional study protocol.

### 2.8 Data collection and management

Pseudonymized data is collected after informed consent is obtained. Questionnaires and sleep log data is collected using paper and pencil and later digitalized under said pseudonymized study code. Sensitive personal data is only accessible by the personnel conducting the study in the specific day clinic and will not be shared between the participating facilities. All digital data including actigraphy is stored under the described study code locally during the duration of the intervention before being anonymized and uploaded to be shared between the participating centers. The upload is conducted to CAU-Cloud, the cloud system provided by the Christian-Albrechts-University in Kiel (CAU), which is secured by the local IT protocols and therefor sufficient to protect study data.

### 2.9 Data analysis

The study is conducted using a control design in which the control group receives regular group therapy. To detect treatment effects, variance in between intervention and control group at T0 and T1 (see Figure 1) is assessed by using a repeated measures ANOVA including two factors (GROUP and TIMEPOINT). Significance level is set to be at *p* = 0.05. Post-hoc tests will clarify effect size and direction. If statistical assumptions are not met, non-parametric alternatives will be used. In additional exploratory analyses we plan to account for sociodemographic aspects or the influence of different diagnoses for the efficacy of the program.

### 2.10 Safety

There are no known risk factors associated with participation. Wearing an actigraph may cause temporary skin irritation or redness similar to wearing a watch. Participants are informed about any risk factors and encouraged to voice concerns regarding their participation during every step of the study. Concerns are documented.

## 3 Discussion

To the best of our knowledge, to this day there is no intervention that targets transdiagnostic sleep problems in children and adolescents with psychiatric disorders in an easily implementable and short group design. This is the case despite extensive evidence demonstration the critical role adequate sleep and sleep quality play in physical and mental wellbeing. Studies have highlighted the negative effects of insufficient sleep on both physical health-such as cardiovascular health and physical fitness (Baranwal et al., 2023; Fonseca et al., 2021)—and even more so psychological health. For example, short sleep duration in young adults is associated with a higher risk to develop or intensify mental disorders such as anxiety, eating disorders, depression or bipolar disorders (Vestergaard et al., 2024). Conversely, adherence to guidelines for physical activity, screen time, and sleep duration has been shown to decrease the risk of the development of mental health problems in children. Notably adapting screen time and ensuring a sufficient sleep duration proved to be the most influential recommendation in prevention of mental health problems in this case (Sampasa-Kanyinga et al., 2020). Furthermore, as published in a recent pre-print by Harvey et al. (2025), variations of the sleep intervention TransS-C in a population with mental health problems significantly reduced sleep disturbances and daytime impairment while overall sleep health was increased. The intervention involving four or more sessions applicable mostly for individual, but also group setting, also improved overall psychiatric symptom severity. We hope to show similar effects while administering a shorter intervention complemented by the use of sleep logs and actigraphy as well as the metaphor tailored to the age of recipients.

Also, in children and adolescents, subjective sleep quality is not only a result of overall mental wellbeing, but there is a clear bidirectional association. Poor self-perceived sleep quality has been shown to increase the risk of anxiety, depression and stress symptoms later on (Zou et al., 2020). Especially in the context of changing sleep characteristics during adolescence, both sleep duration and quality seem to be crucial factors predicting later strain from mental health problems (Thompson et al., 2024; Tarokh et al., 2014). When taking a look at psychiatric samples it is even more evident that sleep problems occur comorbidly with many different psychiatric disorders such as ADHD, autism or depression (van der Heijden et al., 2018; Gupta et al., 2019). Sleep problems may even serve as a common thread linking various psychiatric disorders (Boafo et al., 2021). Diminished sleep quality can also interfere with treatment success of psychotherapy as well as symptom severity at discharge (Buysse et al., 1999; Boafo et al., 2021). Conversely, a reduction of sleep problems helps to improve the outcome of disorder-specific psychotherapy (Freeman et al., 2020). Summarizing the current evidence on the role of sleep in mental wellbeing, and considering the limited resources and options for mental health professionals for addressing sleep problems (Freeman et al., 2020), this underlines recurrent calls for transdiagnostic interventions targeting sleep (Vestergaard et al., 2024; Boafo et al., 2021; Tarokh et al., 2014). This may either be as a form of treatment or as prevention (Boafo et al., 2021). If proven to be efficacious, “Ready for Landing” aims to offer an option to answer those needs by providing a transdiagnostic, economic and easily implementable sleep hygiene intervention. It also seeks to provide knowledge about sleep and sleep hygiene to mental health professionals to sensitize for this matter as advised by Freeman et al. (2020). By improving sleep hygiene, sleep quality, and sleep duration it could therefor positively influence the bidirectional relationship between sleep disturbances and psychiatric disorders and contribute to improved psychological wellbeing in children and adolescents. Some studies propose sleep hygiene interventions to be a first line treatment when targeting sleep problems in the psychiatric context (Baranwal et al., 2023; Chung et al., 2018; Peach et al., 2016), while other studies suggest stand-alone sleep hygiene interventions are lacking substantial treatment effects in the treatment of sleep disorders like insomnia (Irish et al., 2015; Chung et al., 2018). Those studies underscore that if still implemented, sleep hygiene interventions should be done in a standardized and comprehensive delivery of content, rather than relying solely on brochures or psychoeducation. “Ready for Landing” addresses this by following the manual we seek to evaluate here, by incorporating elements from CBT-I, and by helping patients understand the functionality and necessity of sufficient sleep while encouraging them to build routines enabling them to improve their sleep related behaviors (Lin et al., 2018). The combination of CBT-I and its inherent elements of sleep hygiene education has further been proven to be efficacious (Hertenstein et al., 2022). Further by addressing both subjective sleep quality and objectively measured sleep duration the study aims to investigate the effect the intervention has on the parameters known to be influential on mental wellbeing (Vestergaard et al., 2024; Zou et al., 2020). It systematically evaluates the effects as demanded many times when talked about currently available sleep hygiene interventions (Nikles et al., 2020; McLay et al., 2024). If the effects are as proposed, “Ready for Landing” can be seen as an effective, theory-driven intervention to specifically target important domains of generic sleep problems.

### 3.1 Limitations

Even though “Ready for Landing” is based on the current research on sleep interventions, this study as well as the intervention itself have some limitations. Firstly, due to its design, the study does not include a follow-up yet. Although this is planned for future studies, currently the control group receives the intervention at the timepoint T1 (See Figure 1) immediately after the completion of the questionnaires. We use this approach to assure they can benefit from the intervention as well before leaving the day clinic. To further account for the circumstance of frequently changing patient groups, the intervention is limited to only two sessions. This raises the question if this is enough time to thoroughly introduce and establish new sleeping habits. It may be necessary to consider, whether a longer version with more sessions of “Ready for Landing” is necessary to achieve a relevant improvement in sleep. Alternatively, a continuation in individual therapy to further support the implementation of new sleep routines could also be established and evaluated. Another limitation regarding the study design might be the waitlist design which might cause bias, delayed effects and possible confounding effects like seasonal effect regarding light and temperature or holidays. The potential negative influence of the waitlist design should be minimized by the short duration and delay when comparing the conditions.

Second, there is no screening mechanism to rule out if the sleep problems or diminished sleep quality have a somatic cause, such as obstructive sleep apnea or restless legs syndrome. For instance, a study by Andersen et al. (2019) showed that among children and adolescents 9.1% of the participants with a healthy body weight (Body Mass Index Standard Deviation Score (BMI SDS) ≤ 1.28) showed obstructive sleep apnea, while overweight children (BMI SDS > 1.28) even showed a prevalence of 44.6%. A Thai study found that 18.2% of children with ADHD referred to a psychiatric clinic showed signs for high-risk obstructive sleep apnea (Prajsuchanai et al., 2022). These findings suggest potentially higher prevalence of obstructive sleep apnea for children with psychiatric disorders. For those affected, sleep-disordered breathing might partially or completely explain insufficient sleep quality and its influence on overall wellbeing.

Regarding the intervention itself, the following limitations might be taken into consideration: the short and generic character of the intervention does limit its ability to treat, possibly undiagnosed, sleep disorders, such as primary insomnia. Concerning this matter, studies showed that sleep hygiene interventions are less efficacious compared to CBT-I which remains gold standard to treat insomnia (Chung et al., 2018; Deutsche Gesellschaft für Kinder-und Jugendpsychiatrie, Psychosomatik und Psychotherapie e.V. (DGKJP), 2018). Nevertheless, as mentioned above, by incorporating CBT-I elements we hope to further improve efficacy and therefor add a value that exceeds stand-alone psychoeducation, to aim for a significant improvement in sleep quality and elongation of sleep duration. In terms of content, the airplane landing metaphor, which is supposed to illustrate the process of going to sleep, might not help some to understand the underlying context. Not all children and adolescents have experienced a passenger flight themselves. It is essential to ensure that along explaining the metaphor, the sleep hygiene rules are explained clearly as well. Patients more so have to be encouraged to ask questions if the concept remains unclear. Further group therapy format limits the options to discuss specific sleep problems in depth. Participants are invited to talk about their specific and possibly unaddressed sleep problems at the end of the intervention. We further hope increasing awareness about the necessity of restorative sleep might bring sleep and sleep problems into focus, which are then addressed in individual therapy if needed. Concerning the setting, it also has to be discussed on how to transfer “Ready for Landing” to different settings such as individual or inpatient treatment. Especially with inpatient treatment it has to be examined, if rules, such as only using the bed to sleep, are possible to implement in the current layout of treatment facilities. In those more restrictive environments, there is a need to come up with treatment concepts that enable a good sleep hygiene and that ensure patients can autonomously build those new habits.

### 3.2 Further directions

If proven efficacious, we aim to provide “Ready for Landing”—including its manual and materials—available for implementation by other professionals. Furthermore, we aim to examine the intervention’s feasibility in other contexts than day clinic treatment, such as inpatient or outpatient treatment groups, or educative programs with healthy youth. Potentially we could further aim to investigate its efficacy regarding specific disorders and explore different age groups.

## 4 Conclusion

In conclusion the study aims to evaluate the efficacy of a sleep hygiene intervention for children and adolescents with psychiatric disorders, on both, objective and subjective measures of sleep quality, and sleep duration. “Ready for Landing” conveys the principles of sleep hygiene through the metaphor of a landing passenger airplane, describing a well-structured process. The intervention consists of different phases and uses psychoeducative as well as cognitive-behavioral elements. The program is developed to be implemented as a group therapy-based intervention with two sessions for children and adolescents aged 10–18 years in day clinic treatment. It is hypothesized that the intervention is capable to improve sleep quality and elongate sleep duration. If “Ready for Landing” shows to be efficacious, it could offer a simple and cost-effective treatment option to help bridge the gap between highly prevalent sleep problems in children and adolescents suffering from mental health problems and the often-unmet therapeutic needs.
