# Supplementary material for: “Ready for Landing”—study protocol for the evaluation of a brief sleep hygiene group intervention for youth with psychiatric disorders
Source: Front Psychol. 2025 Aug 14;16:1543448. doi: 10.3389/fpsyg.2025.1543448 (PMC12392276; doi:10.3389/fpsyg.2025.1543448)
Supplement: Supplementary file 1 [file Data_Sheet_1.pdf]

SPIRIT

STANDARD PROTOCOL ITEMS: RECOMMENDATIONS FOR INTERVENTIONAL TRIALS

SPIRIT 2013 Checklist: Recommended items to address in a clinical trial protocol and related documents*

Section/item	Item No	Description
**Administrative information**		
Title	1	"Ready for Landing" – study protocol for the evaluation of a brief sleep hygiene group intervention for youth with psychiatric disorders
Trial registration	2a	German Clinical Trials Register<br>Identifier: DRKS00034984<br>(https://www.bfarm.de/Deutsches-Register-Klinischer-Studien.html)
	2b	Not applicable, registered at DRKS
Protocol version	3	Version 1, 13.09.2024
Funding	4	"Ready for Landing" has been developed as part of "Res@t (Resource-Strengthening Training for Adolescents with Problematic Digital-Media Use and their Parents)" [41] .This project received funding by the German Innovation Fund of The Federal Joint Committee (G-BA Innovationsfond: 01NVF20011) the Open Access Publication Fund of University Medical Center Hamburg-Eppendorf (UKE) and the German Research Foundation (DFG). "Ready for Landing" itself did not receive any financial funding. The work of PTM is supported by a grant of the German Research Foundation (DFG, project nr.: 468645090). The funding sources did not influence the design of the study nor will they have influence on its execution or data analysis.<br><br>"Ready for Landing" therefor also did not undergo peer-review yet.

Roles and responsibilities	5a	Paula Theresa Meyer1*, Hannah Brauer1, Clara Marie Schreiber2, Pia Muriel Heinze1, Julia Witte3,4, Christoph Berger3,4, Manuel Munz1,2, Alexander Dück3,4, Olaf Reis3,4, Michael Kölch3,4, Alexander Prehn-Kristensen1,5<br>1. Institute of Child and Adolescent Psychiatry, Center for Integrative Psychiatry, School of Medicine, Christian-Albrecht University Kiel, D-24105 Kiel, Germany.<br>2. Clinic for Child and Adolescent Psychiatry, Psychotherapy and Psychosomatics, Center for Integrative Psychiatry, School of Medicine, D-24105 Kiel, Germany<br>3. Department for Child and Adolescent Psychiatry and Neurology, Rostock University Medical Center, D-18147 Rostock, Germany<br>4. German Center for Child and Adolescent Health (DZKJ), partner site Greifswald/Rostock, site Rostock, D-18147 Rostock, Germany<br>5. Department of Psychology, Faculty of Human Sciences, MSH Medical School Hamburg - University of Applied Sciences and Medical University, D-20457 Hamburg, Germany<br><br>APK, HB and CMS developed the intervention. HB, PMH, JW and PTM prepared materials and organized the implementation of the study. AD, CB, OR, JW and PTM developed the regimen to conduct actigraphy. APK, MM, OR and MK supervise the study. HB & PTM wrote the manuscript, all authors reviewed the manuscript and agreed on publishing it.<br><br>PTM is corresponding author.
	5b	Not applicable
	5c	Not applicable
	5d	Not applicable

Introduction

Background and rationale	6a	People with mental disorders frequently exhibit comorbid sleep problems. This is also true for children and adolescents in psychiatric treatment. Their sleep problems interact with the course and treatment outcomes of their mental disorders. This study aims to evaluate a brief sleep hygiene intervention for children and adolescents in day clinics. The intervention, 'Ready for Landing,' is a group therapy program consisting of two sessions, where children and adolescents are taught the basics of sleep hygiene using the metaphor of an airplane landing. The intervention incorporates cognitive-behavioral elements to improve sleep quality and increase sleep duration. The sessions include reflecting on one’s own sleep and sleep hygiene, as well as applying and reinforcing sleep hygiene rules. The study is conducted using a waitlist control group design. Participants in the intervention group first receive the group therapy intervention over two sessions, followed by a session of regular group therapy without sleep-related content. Participants in the control group initially receive a session of regular group therapy without sleep-related content, followed by the intervention. Group assignment is allocated pseudo-randomized. Group affiliation is determined by the timing of the condition being implemented. The intervention group and control group are alternated with sufficient time intervals, allowing the patient population within the facilities to change in the meantime.Subjective sleep parameters are assessed before the sessions using sleep questionnaires. All participants keep a sleep diary throughout the study period. To measure objective sleep parameters, sleep and wake times are recorded using actigraphy. We expect the intervention to have positive effects on both subjective and objective sleep quality and sleep duration.<br>For literature see:<br>• Freeman D, Sheaves B, Waite F, Harvey AG, Harrison PJ. Sleep disturbance and psychiatric disorders. Lancet Psychiatry. 2020;7:628–37. doi:10.1016/S2215-0366(20)30136-X.<br>• Reynolds KC, Alfano CA. Childhood Bedtime Problems Predict Adolescent Internalizing Symptoms Through Emotional Reactivity. J Pediatr Psychol. 2016;41:971–82. doi:10.1093/jpepsy/jsw014.<br>• Baglioni C, Nanovska S, Regen W, Spiegelhalder K, Feige B, Nissen C, et al. Sleep and mental disorders: A meta-analysis of polysomnographic research. Psychol Bull. 2016;142:969–90. doi:10.1037/bul0000053.<br>• Baglioni C, Battagliese G, Feige B, Spiegelhalder K, Nissen C, Voderholzer U, et al. Insomnia as a predictor of depression: a meta-analytic evaluation of longitudinal epidemiological studies. J Affect Disord. 2011;135:10–9. doi:10.1016/j.jad.2011.01.011.<br>• Inada K, Enomoto M, Yamato K, Marumoto T, Takeshima M, Mishima K. Effect of residual insomnia and use of hypnotics on relapse of depression: a retrospective cohort study using a health

		insurance claims database. J Affect Disord. 2021;281:539–46. doi:10.1016/j.jad.2020.12.040.<br><br>• Tarokh L, Hamann C, Schimmelmann BG. Sleep in child and adolescent psychiatry: overlooked and underappreciated. Eur Child Adolesc Psychiatry. 2014;23:369–72. doi:10.1007/s00787-014-0554-7.<br>• Boafo A, Dion K, Greenham S, Barrowman N, Reddy D, Koninck J de, Robillard R. Sleep problems and complexity of mental health needs in adolescent psychiatric inpatients. J Psychiatr Res. 2021;139:8–13. doi:10.1016/j.jpsychires.2021.05.005.<br>• Hertenstein E, Trinca E, Wunderlin M, Schneider CL, Züst MA, Fehér KD, et al. Cognitive behavioral therapy for insomnia in patients with mental disorders and comorbid insomnia: A systematic review and meta-analysis. Sleep Med Rev. 2022;62:101597. doi:10.1016/j.smrv.2022.101597.<br>• Gupta P, Sagar R, Mehta M. Subjective sleep problems and sleep hygiene among adolescents having depression: A case-control study. Asian J Psychiatr. 2019;44:150–5. doi:10.1016/j.ajp.2019.07.034.<br>• van der Heijden KB, Stoffelsen RJ, Popma A, Swaab H. Sleep, chronotype, and sleep hygiene in children with attention-deficit/hyperactivity disorder, autism spectrum disorder, and controls. Eur Child Adolesc Psychiatry. 2018;27:99–111. doi:10.1007/s00787-017-1025-8.
	6b	The comparator was chosen to minimize influence on regular treatment process and to ensure both groups receive the intervention.
Objectives	7	The study aims to evaluate the efficacy of a group therapy-based sleep hygiene intervention in day clinic treatment for children and adolescents with psychiatric disorders. We expect to improve sleep quality and elongate sleep duration in children and adolescents with mental health problems by administrating “Ready for Landing” (RfL). On an exploratory level we aim to investigate the effect the intervention has on the sleep onset latency and sleepiness.
Trial design	8	The study is a non-randomized controlled study with an intervention and a control group.<br>Group affiliation is determined quasi randomized by time. Intervention and control group are carried out alternating about eight to ten weeks apart.

Methods: Participants, interventions, and outcomes

Study setting	9	The study is carried out in day clinics for child and adolescent psychiatry in Germany. Study sites can be found here: https://drks.de/search/en/trial/DRKS00034984
Eligibility criteria	10	The study includes children and adolescents of all genders ranging from 10 to 18 years of age that undergo psychiatric treatment in a participating day clinic. Exclusion criteria for the study are the end of treatment and insufficient knowledge of the german language.
Interventions	11a	The intervention group will receive the first session of the intervention after giving informed consent and completing a 7-day baseline measurement of actigraphy at time point t0. After another week, at t1, the intervention group will receive the second session of the brief intervention. One week later, at t2, the intervention group will receive standard group therapy program. The actigraphy measurement will end one week after t2. The intervention explains sleep hygiene by using the metaphor of a landing airplane. It incorporates psychoeducative elements as well as techniques from cognitive behavioural therapy.<br>The control group will initially receive a standard group therapy program after giving informed consent and completing a 7-day baseline measurement of actigraphy at time point t0. After another week, they will receive the first session of the intervention at t1. One week later, at t2, the control group will receive the second session of the intervention. The actigraphy measurement will end one week after t2. The content of the intervention does not differ.
	11b	Not applicable
	11c	Not applicable
	11d	No restrictions
Outcomes	12	Primary outcome:<br>Primary outcome of the study is the change in sleep quality (measured with the PSQI) and in sleep duration (Total Sleep Time (TST) measured with actigraphy) from baseline to post measurement.<br>Secondary outcome:<br>Secondary outcomes of the study are subjectively perceived sleep duration and sleep onset latency (assessed using sleep logs) and sleepiness (assessed using ESS-CHAD).<br>For the PSQI a reduced sum score is proposed to show a reduction in symptom severity. Same thing is anticipated for the sum score of the ESS-CHAD. Treatment effect on sleep duration is to be measured as an elongated sleep duration assessed with actigraphy. We do expect no possible harm outcomes during the study and therefor did not define specific indicators.

Participant timeline	13	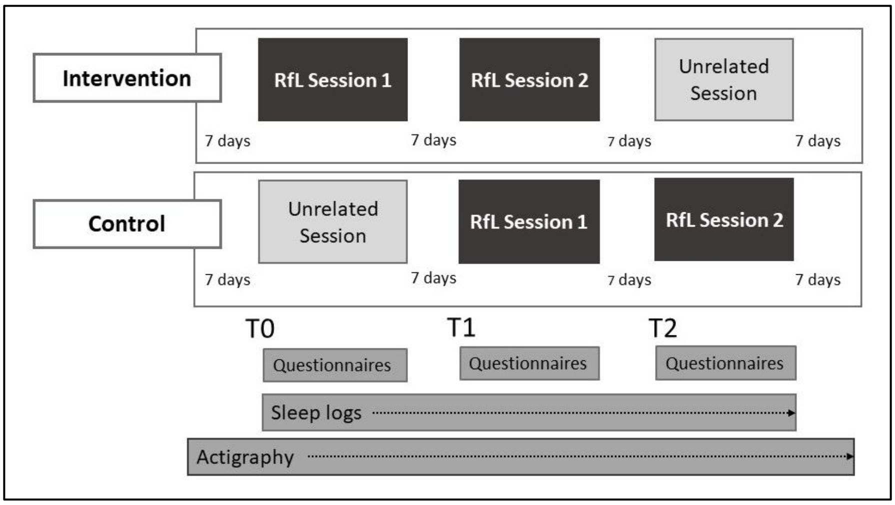<br><br>The study starts with obtaining informed consent from both children/adolescents and their parents/custodians. The further procedure is depicted in the figure.
Sample size	14	The sample size was computed assuming a repeated measures ANOVA with a within-between interaction using g*Power 3.1. We assumed a middle effect size (d = .25), a power of .90 and an alpha-error of 5%. This resulted in a minimum sample size of N = 46 participants. As we expect a drop-out rate of about 25%, we calculate with 60 participants to include into the study.
Recruitment	15	Participants are recruited out of patients admitted to the facilities. To ensure achieving adequate sample size time frames for the cycles of the intervention are set by personnel overseeing new admissions and releases.

Methods: Assignment of interventions (for controlled trials)

Allocation:

Sequence generation	16a	Not applicable
Allocation concealment mechanism	16b	Not applicable
Implementation	16c	Not applicable
Blinding (masking)	17a	There will be no blinding during the study.
	17b	Not applicable

Methods: Data collection, management, and analysis

Data collection methods	18a	To measure subjective changes in sleep quality we use the Pittsburgh Sleep Quality Index (PSQI). The PSQI consists of 24 Items regarding seven domains of sleep quality including sleep quality, sleep latency, sleep duration, sleep efficiency, sleep disturbances, medication use, and daytime sleepiness. The PSQI is a validated and widely used self-report measure for sleep quality showing good reliability, internal consistency, criterion validity and discriminatory power. A cut-off score of five has been deemed sufficient to discriminate between good and bad sleep quality. Those findings have been replicated for the use of the PSQI in minors. To assess daytime sleepiness, we use the Epworth Sleepiness Scale for Children and Adolescents (ESS-CHAD) which consists of eight items regarding the tendency to fall asleep during different age-adjusted daytime activities such as during lessons in school. The ESS-CHAD has been shown to be a reliable and valid measure to assess daytime sleepiness in minors. PSQI and ESS-CHAD are distributed at the beginning of every session. Between sessions the patients keep a sleep log that monitors aspects such as bedtime, waketime, total sleep duration, duration until they fall asleep and subjective aspects such as perceived rest- and wakefulness each day during the week. Those subjective measures will be complemented by actigraph data to gain insides about possible incremental validity. This part of the study will be described in an additional protocol.<br>In this study actigraph data will only be used in terms of assessing treatment effects on sleep duration (Total Sleep Time, TST). Therefor the MotionWatch 8 (Alpha Trace Ltd.) a wrist worn actigraphy watch worn continuously throughout the 28 day will be used. We will use MotionWatch Mode 1 and record 30 second epochs of movement and light information.
	18b	Not applicable
Data management	19	During the study case report forms for each participant are obtained. Data, each paper pencil and actigraphy, is stored pseudonymized aligned to local data safety regulations as proposed by the DSGVO. Data storage takes place on secure computers in the facilities and secured lockers for personal information such as names and addresses.

Statistical methods	20a	To detect a treatment effect, variance in between intervention and control group at T0 and T1 (see Fig. 1) is assessed by using a repeated measures ANOVA including two factors (GROUP and TIMEPOINT). Significance level is set to be at p = 0.05. Post-hoc tests are going to be used to determine direction and size of possible effects. In case statistical assumptions necessary for the rmANOVA are not met it is going to be defaulted on non-parametric alternatives. In additional exploratory analyses we plan to account for sociodemographic aspects or the influence of different diagnoses for the efficacy of the program.
	20b	See above.
	20c	Not applicable
**Methods: Monitoring**		
Data monitoring	21a	Data monitoring takes place in the participating facilities. No additional monitoring through sponsors etc. from the outside takes place.
	21b	Not applicable, study will be conducted until the predefines sample size is met.
Harms	22	Harms are not expected to occur throughout the study. Nevertheless participants are encouraged to voice any concerns and direct them towards the responsible personnel in their facility.
Auditing	23	Not applicable.
**Ethics and dissemination**		
Research ethics approval	24	The study has been approved by the ethics committee of both the medical faculty of the Christian-Albrechts-University in Kiel and the medical faculty of the Rostock University Medical Center. The study will be conducted in line with institutional requirements and local legislation. Publications regarding the study will be written in collaboration of the participating facilities. The trial is registered on the German Clinical Trials Register (DRKS00034984, registered on 13.09.2024, https://www.bfarm.de/Deutsches-Register-Klinischer-Studien.html).
Protocol amendments	25	Not applicable
Consent or assent	26a	Informed Consent is obtained by mental health professionals within the facilities. Participants are informed about the study procedure, so are their parents/custodians in case they are younger than 18. Data collection starts after written IC is obtained.
	26b	Not applicable

Confidentiality	27	Personal information will be handled confidential. All study data is stored pseudonymized. Personal information will not be shared between facilities or with others. Personal data is stored for 10 years according to local regulations and will then be destroyed.
Declaration of interests	28	APK received research funding and HB consulting fees by mementor by ResMed. All other authors declare that the research was conducted in the absence of any commercial or financial relationships that could be construed as a potential conflict of interest.
Access to data	29	Data will be analysed by the scientific staff in the facilities. Data transfer between facilities will be anonymized and takes place via cloud services provided by the university in Kiel and is therefor deemed safe.
Ancillary and post-trial care	30	Not applicable
Dissemination policy	31a	Results of the study will be published scientificly together by the authors. The authors therefor agree on cooperating work doing so.
	31b	Not applicable
	31c	Data and information can be shared upon reasonable request.
**Appendices**		
Informed consent materials	32	See attached.
Biological specimens	33	Not applicable

*It is strongly recommended that this checklist be read in conjunction with the SPIRIT 2013 Explanation & Elaboration for important clarification on the items. Amendments to the protocol should be tracked and dated. The SPIRIT checklist is copyrighted by the SPIRIT Group under the Creative Commons "Attribution-NonCommercial-NoDerivs 3.0 Unported" license.
